# Exosomal miR-17-5p from human embryonic stem cells prevents pulmonary fibrosis by targeting thrombospondin-2

**DOI:** 10.1186/s13287-023-03449-7

**Published:** 2023-09-04

**Authors:** Qun Liu, Youkun Bi, Shaole Song, Keqi Zhu, Xinlong Qiao, Huiwen Wang, Guangju Ji

**Affiliations:** 1 Institute of Biophysics, Chinese Academy of Sciences, Beijing, 100101 China; 2 University of Chinese Academy of Sciences, Beijing, 100049 China

**Keywords:** Pulmonary fibrosis, Human embryonic stem cell-derived exosomes, Bleomycin, miR-17-5p, Thbs2

## Abstract

**Background:**

Idiopathic pulmonary fibrosis (IPF) is a chronic, progressive, and irreversible lung disease characterized by pulmonary fibrosis and lung dysfunction, ultimately leading to respiratory failure. Many preclinical studies have investigated the therapeutic potential of stem cell-derived exosomes in this disease, particularly mesenchymal stem cell-derived exosomes. However, the effects of embryonic stem cell-derived exosomes in IPF remain unclear.

**Methods:**

We established a bleomycin (BLM)-induced pulmonary fibrosis mice model and administered human embryonic stem cell exosomes (hESC-exo) from the first day after BLM treatment. The effects of hESC-exo were assessed by pulmonary function tests, biochemical analysis, histochemistry, quantitative real-time polymerase chain reaction (qPCR), and western blot (WB). RNA-seq was used to screen for the potential therapeutic targets of hESC-exo in fibrotic lungs; the identified signaling axis was characterized using a luciferase assay, qPCR, and WB.

**Results:**

Results indicated hESC-exo administration notably alleviated inflammation, removed deposited collagen, and rescued alveolar architecture in the lungs of BLM-induced mice. In vivo and in vitro tests revealed that hESC-exo-derived miR-17-5p directly bound thrombospondin-2 (Thbs2) to regulate inflammation and fibrosis; thus, hESC-exo protected against BLM toxicity in the lungs via the miR-17-5p/Thbs2 axis.

**Conclusion:**

These results suggest a promising new treatment for fibrosis-associated diseases.

**Supplementary Information:**

The online version contains supplementary material available at 10.1186/s13287-023-03449-7.

## Introduction

Idiopathic pulmonary fibrosis (IPF) is a chronic, progressive, and irreversible lung disease characterized by inflammatory cell infiltration, pulmonary fibroblast proliferation, and extracellular matrix (ECM) collagen deposition, leading to pulmonary fibrosis and lung dysfunction [1, 2]. Lung transplantation is the optimal option for end-stage patients, but is limited by scarce donors [3, 4]. The approved pharmaceutical strategies, nintedanib, and pirfenidone, attenuate the progression of pulmonary fibrosis, but still fail to improve survival rates [5]. Due to the low average survival time (approximately 3–5 years) and poor quality of life, there is an urgent need to develop novel and effective treatment strategies against this disease [6–8].

Bleomycin (BLM) is an anticancer drug used to treat various types of neoplasms [9]. Its most adverse effect is lung toxicity, causing collapse of the normal lung architecture and attenuation of lung function. In many preclinical studies, BLM is one of the most widely used drugs for inducing lung fibrosis in rodents, on account of its ability to induce a histological lung pattern similar to that described in patients undergoing chemotherapy [9, 10]. BLM-induced lung fibrosis is characterized by regional inflammation, epithelial cell dysplasia, epithelial-mesenchymal transition, myofibroblast activation, and injuries to the basement membrane and alveolar epithelium. Studies have demonstrated that BLM induces oxidative damage and DNA strand breakage, with early pathological manifestations of exudative alveolitis, generation of inflammation, late pathological manifestations of stromal cell proliferation, and matrix collagen aggregation [10, 11].

To date, the mechanisms involved in the onset and development of pulmonary fibrosis have not been thoroughly clarified. Two signaling pathways, the inflammatory and epithelial pathways, dominate fibrogenesis in the lung [12, 13]. Stress-activated fibroblasts secrete profibrotic cytokines, e.g., transforming growth factor β (TGF-β), to stimulate fibroblastic overexpression of fibrotic matrix and further induction of profibrotic cytokine expression, leading to progressive fibrosis. Moreover, profibrotic cytokines activate epithelial cells, which have functions and protein expression patterns that overlap with those of activated fibroblasts. This vicious circle contributes greatly to the development of severe fibrosis [14]. At the pathological level, the increasing ECM destroys normal alveolar architecture and disrupts gas exchange, which impairs lung function. In particular, abnormal activation of the TGF-β signaling pathway triggers fibrogenesis by recruiting the downstream transcription factor, Smad2/3, and this signaling is crucial throughout the progression of fibrosis [15, 16].

According to numerous preclinical investigations, stem cell-based therapies have a broad application in pulmonary fibrosis, particularly mesenchymal stem cells (MSCs), e.g., bone, umbilical cord, and adipose-derived MSCs [17–19]. However, there are some thorny issues hindering the large-scale clinical application of these therapies, such as low survival rates, immunological rejection, and safety issues [20]. With the exponential increase in vesicle research, stem cell-derived exosomes have become a safer and more effective alternative for the treatment of lung damage, reliant on their bioactive cargoes [21]. Some of their advantages have accelerated preclinical trials of pulmonary fibrosis; these advantages include improved biosecurity and stability, non-aneuploidy, and low immunogenicity. Previous studies have reported that MSC-derived exosomes (MSC-exo) alleviate lung fibrosis by promoting the regeneration of alveolar epithelial cells, inhibiting the inflammatory response, and repressing the activation of myofibroblasts, that facilitate the removal of collagen deposits and restoration of alveolar structure [22, 23].

To date, much attention has been paid to MSC-exo on account of the broader clinical application of MSCs, while embryonic stem cell-derived exosomes (ESC-exo) have been snubbed due to their tumorigenicity and the ethical issues surrounding parental cells. Nonetheless, some studies have investigated the role of ESC-exo in regulating systemic aging and inflammation by delivery of bioactive cargoes [24, 25]. Given the positive therapeutic effects of ESC-exo in the repair of tissue damage, they should have the potential to treat lung damage such as pulmonary fibrosis. To test this hypothesis, we isolated exosomes from human ESCs (hESC-exo), investigated their effects in BLM-induced pulmonary fibrosis, and further clarified the underlying mechanisms.

## Materials and methods

### Animals

Eight-week-old C57BL/6 male mice (Beijing Sipeifu Biological Co., Ltd.) were kept in separate cages and given food and water ad libitum. Mice were housed under controlled temperature (22 ± 2 °C) and relative humidity (40–60%) conditions with a 12-h light/dark cycle. All procedures on mice were performed in adherence with the Guide for the Care and Use of Laboratory Animals published by the U.S. National Institutes of Health (NIH Publication No. 85–23, revised 1996) and with the approval of the Institute of Biophysics Committee for Animal Care (Approval No. SYXK2020053).

### Cell culture

Beas-2b and 293 T cell purchased from ATCC were cultured in Dulbecco’s Modified Eagle’s Medium (DMEM) with high glucose (DMEM/HD) supplemented with 10% fetal bovine serum (FBS), 100 U/mL penicillin, and 100 μg/mL streptomycin, in an incubator with 5% CO_2_ at 37 °C. When confluence reached 85%, cells in good condition were passaged at a ratio of 1:3. The human embryonic stem cell (hESC) line H9 was kindly provided by the Stem Cell Bank, Chinese Academy of Sciences (Beijing, China). H9 cells were cultured in Nuwacell™ ncTarget medium (Cat. No. RP01020, Nuwacell Biotechnologies, China) supplemented with penicillin, streptomycin, and amphotericin and grown in dishes coated with vitronectin (Cat. No. RP01002, Nuwacell Biotechnologies), at 37 °C and 5% CO_2_. H9 cells were passaged at a ratio of 1:20 every four days using Nuwacell™ hPSC Dissociation Buffer (Cat. No. RP01007, Nuwacell Biotechnologies).

### Isolation and identification of exosomes

Exosomes were extracted from collected hESC supernatant using differential centrifugation methods, as previously described [26]. Briefly, the supernatant was centrifuged at 300 × *g* for 10 min, the resulting supernatant centrifuged again at 2000 × *g* for 10 min, the supernatant was collected and centrifuged at 10,000 g for 20 min, and the supernatant from this step once more centrifuged at 100,000 × *g* for 70 min using an ultracentrifuge (Optima XPN-100, Beckman Coulter, Germany). The resulting pellet was washed with PBS, centrifuged at 100,000 × *g* for 70 min, then dissolved in PBS, and stored at − 80 °C until use. Exosome morphology was observed using a transmission electron microscope (Tecnai Spirit 120 kV, FEI, USA) after negative staining. Exosome diameters were measured by dynamic light scattering (271-DPN, Wyatt Technology, USA), and exosomal surface markers CD63 (1:1000; Cat no. ab217345, Abcam, USA), CD9 (1:1000; Cat no. ab236630, Abcam) and Tsg101 (1:1000; Cat. No. ab125011, Abcam) were analyzed by western blot. The protein concentrations of hESC-exo were measured using a Bradford protein concentration assay kit; dosages for all subsequent experiments were based on these measured protein concentrations.

### In vivo tracking of hESC-exo

Isolated exosomes were stained with PKH67 (Cat. No. MINI67, Sigma, USA), according to the manufacturer’s instructions, mixed hESC-exo with PKH67 in diluent C for 4–10 min in the dark, terminated the staining reaction with 0.5% bovine serum protein (BSA)/PBS, and centrifuged at 10,000 × *g* for 70 min, followed by resuspension of the pellet in PBS. Mice were administered dye-labeled hESC-exo via tail vein injection. Mouse lungs were harvested and imaged using the IVIS Lumina III In Vivo Imaging System (Caliper, USA) at 24 h, 48 h, and 72 h post-injection.

### Animal treatment with bleomycin and hESC-exo

A total of seventy-two 8-week-old male C57BL/6 mice were included in the experiment. 60 mice were treated with 3 mg/kg BLM (Cat. No. MB1039, Meilunbio, China) in 50 μL NS on day 0 via intratracheal injection. Subsequently, BLM-treated mice were randomly divided into two groups: BLM + exo (20 mice, administered 20 µg hESC-exo in 200 µL PBS every two days), and BLM + PBS (20 mice, administered 200 µL PBS) groups. Random numbers were generated using the standard = RAND () function in Microsoft Excel. The control group consisted of mice were administered with NS and PBS using the same route and volume parameters as the other groups (8 mice, defined as the NS + PBS group). All experimental mice were monitored daily for 7 or 21 days for weight and mortality. At the experimental endpoint, mice were killed by CO_2_ asphyxiation; the BALF and lungs were harvested shortly afterward for subsequent analysis.

To investigate the therapeutic effect of hESC-exos on pulmonary fibrosis model, the fibrosis mice induce by BLM for 20 days were administrated with hESC-exos (8 mice, 20 µg hESC-exo in 200 µL PBS) or selfsame volume PBS (8 mice), twice a week for 5 weeks. The control group consisted of mice were administered with NS and PBS using the same route and volume parameters as the other groups (4 mice, defined as the NS + PBS group). Mice that lost less than 10% of their body weight prior to hESC-exos injection were considered unsuccessful in modeling and excluded from the experiment (4 mice were excluded). At the endpoint, mice were killed and subjected to pathological histology analysis.

### Establishment of a BLM toxicity cell model and hESC-exo intervention

Beas-2b cells grown in 6-well-plates, at 80% confluence, were treated with DMEM containing 10% FBS and 1 μg/mL BLM. Subsequently, BLM-treated cells were treated with 2 μg/mL hESC-exo or equal volume of PBS. After 24 h, cells were digested and pelleted for protein preparation and RNA isolation, as previously described. Cells treated with an equal volume of PBS were used as the control group.

### Histopathology staining

Harvested lungs were fixed with 4% paraformaldehyde for 48 h. Selected tissues were embedded in paraffin after dehydration in 75% ethanol for 12 h, and then cut into 5 μm sections. Tissues were deparaffinized using a standard xylene-deparaffin procedure and then subjected to hematoxylin and eosin (H&E) staining, Masson’s trichrome stain, and Sirius red stain, as previously described. Statistics of collagen area based on Masson’s trichrome stain was performed by an experienced blinded pathologist who is unaware of the experimental design.

For immunohistochemical analysis, antigen retrieval was performed after dewaxing and rehydration. Sections were then incubated with primary antibodies: anti-Collagen I (1:400; Cat. No. PA1-26,204, ThermoFisher, USA) and Fibronectin (1:250; Cat. No. ab2413, Abcam), at 4 °C for 16 h after blocking the tissue with blocking solution for 1 h. Sections were then washed, before incubation with biotin-conjugated secondary antibodies for 1 h at room temperature. Following incubation with streptavidin–biotin complex-horseradish peroxidase (HRP) (Cat. No. P0615, Beyotime, China) for 40 min, sections were stained with 3,3’-diaminobenzidine (Cat. No. P0203, Beyotime).

### Enzyme-linked immunosorbent assay

IL-6, IL-1β, and IL-17A levels in bronchoalveolar lavage fluid (BALF) were determined using commercial enzyme-linked immunosorbent assay (ELISA) kits (Cat. No. KE10007, KE20005, and KE10020, respectively, Proteintech, China) according to the manufacturer’s instructions. In brief, samples or standards were added to the appropriate wells of a precoated microplate, and incubated for 120 min at 37 °C. After three washes, biotinylated detection antibody was added to wells for 60 min at 37 °C, followed by reaction with streptavidin-HRP for 40 min at 37 °C. After a further wash, 100 µL tetramethylbenzidine substrate was added to each well for 30 min at 37 °C. The reaction was stopped with 50 µL 0.5 M H_2_SO_4_, and OD_450_ and OD_630_ of each well were immediately read using a microplate absorbance reader (Bio-Rad, USA).

### Hydroxyproline content measurement

Hydroxyproline (HYP) content was measured using an HYP content detection kit (#BC0250, Solarbio, China) according to the manufacturer’s instructions. Lung tissue was homogenized and mixed with extraction solution for 2 h at 110 °C. The pH of samples was adjusted to 6–8, and the volume made up to 5 mL with distilled water. The supernatant was collected after centrifugation at 12,000 × *g* for 20 min, and the absorbance measured at 560 nm.

### Pulmonary function measurement

Awake mice were subjected to pulmonary function assessment using a FinePointe Non-Invasive Airway Mechanics system (Leica, Germany). Mice in their natural state were placed into a calibrated chamber for the first adaptive respiratory activity monitoring session. After the adaptation measurement, breath activities were recorded in real-time, including the respiratory frequency (breaths per minute; BPM), tidal volume (TV), lung minute volume (LMV), and airway resistance (AR). Three independent replicates were performed for each trial.

### CT screen

Mice were anesthetized with isoflurane (Cat. No. R510-22-8, RWD, China) and then immobilized on the support platform in a supine position for tomography imaging using an in vivo living imaging system (Quantum FX, PerkinElmer, USA). A CT scan of the lung, with a period of 4.5 min, was performed at a voltage of 90 kV and a current of 80 μA.

### Electron microscopy

Fresh lung tissue was cut into 0.1 × 0.1 × 0.1 cm sections and immediately immersed into 2.5% glutaraldehyde solution for 48 h fixation. Sections were then fixed with osmium, dehydrated with an alcohol gradient, and infiltrated with an embedding agent, before cutting into ultra-thin slices of 60–80 nm. Slices were then stained with uranium acetate and lead citrate. Stained slices were observed under a transmission electron microscope (Spirit 120 kV, FEI, USA).

### Pulmonary blood flow monitoring

Pulmonary blood flow was monitored using an RFLSI III laser speckle contrast imaging system (RWD). Briefly, mice were placed on the operating table after induction of anesthesia, and breathing was maintained with a small animal ventilator. Mouse chests were opened to expose lungs to the imaging system laser, and blood flow changes in the lungs were then recorded.

### Quantitative real-time PCR

Total RNA was extracted by the TRIzol (Cat. No. RN0102, Aidlab Biotechnologies, China) method, as previously described. It was then reverse transcribed to cDNA using a PrimeScript RT Reagent Kit (Cat. No. RR047A, TaKaRa, China) and miRNA First Strand cDNA Synthesis Kit (Cat. No. B532451, Sangon, China), according to the manufacturers’ protocols. A qPCR assay was performed using a SYBR Premix Ex Taq II Kit (Cat. No. RR820DS, TaKaRa), and the expression levels of RNA and microRNA were normalized to the levels of β-actin and U6. Primers used in the qPCR assay are listed in Tables 1 and 2.

**Table 1:** mRNA primer pairs used in recent study

Name	Forward primer	Reverse primer
mIL-6	CTCCCAACAGACCTGTCTATAC	CCATTGCACAACTCTTTTCTCA
hIL-6	AGACAGCCACTCACCTCTTCAG	TTCTGCCAGTGCCTCTTTGCTG
mIL-1β	TGAAATGCCACCTTTTGACAG	CCACAGCCACAATGAGTGATAC
hIL-1β	CCACAGACCTTCCAGGAGAATG	GTGCAGTTCAGTGATCGTACAGG
mIL-17A	TGTGAAGGTCAACCTCAAAGTC	AGGGATATCTATCAGGGTCTTCATT
hIL-17A	CGGACTGTGATGGTCAACCTGA	GCACTTTGCCTCCCAGATCACA
mTGFβ1	GCAACAATTCCTGGCGTTACCTTG	CAGCCACTGCCGTACAACTCC
hTGFβ1	TACCTGAACCCGTGTTGCTCTC	GTTGCTGAGGTATCGCCAGGAA
mα-SMA	GTACCCAGGCATTGCTGACA	GAGGCGCTGATCCACAAAAC
hα-SMA	CTATGCCTCTGGACGCACAACT	CAGATCCAGACGCATGATGGCA
mFn	GCAAGAAGGACAACCGAGGAAA	GGACATCAGTGAAGGAGCCAGA
hFn	ACAACACCGAGGTGACTGAGAC	GGACACAACGATGCTTCCTGAG
mCollagen I	GTCAGACCTGTGTGTTCCCTACTCA	TCTCTCCAAACCAGACGTGCTTC
hCollagen I	GATTCCCTGGACCTAAAGGTGC	AGCCTCTCCATCTTTGCCAGCA
mThbs2	TCGGACCTCAAGTATGAGTGC	ATCTAAGAAGGGGTGTTTGCAG
hThbs2	CAGTCTGAGCAAGTGTGACACC	TTGCAGAGACGGATGCGTGTGA
mCol11a1	GGAAAGATGGGCTACCAGGACA	TAGGACCAGTCTCACCAGTTGG
mSOX9	TCCTGCAGAGAGACATCGGA	GAAAGGCAGGGTGCACAAAG
mSiglec1	TCTCGGCTCCTGTGGTCCTAAG	TCCACAGTGCAGATGAACACGG
mDchs2	ATGCTGGCCCTAAACCAGAC	CAGCAAGAATGATGAGCCGC
mPrf1	ACACAGTAGAGTGTCGCATGTAC	GTGGAGCTGTTAAAGTTGCGGG
mColq	CCTCCAGGAAGATGCCTTTGCG	CAAGCTCTTCCTGGTTGTTGACC
mβ-actin	GGACTGTTACTGAGCTGCGTT	CGCCTTCACCGTTCCAGTT
hβ-actin	CACCATTGGCAATGAGCGGTTC	AGGTCTTTGCGGATGTCCACGT

*m*: mouse; *h*: human; *IL*: interleukin; *TGF*: transforming growth factor; *α-SMA*: alpha-smooth muscle actin; *Fn*: fibronectin; *Thbs2*: thrombospondin-2; *Col11a1*: collagen type XI alpha 1 chain; *Sox9*: SRY-Box transcription factor 9; *Siglec1*: sialic acid binding Ig like lectin 1; *Dchs2*: dachsous cadherin-related 2; *Prf1*: perforin 1; *Colq*: collagen-tail subunit of acetylcholinesterase

**Table 2:** microRNA primers used in recent study

Name	Forward primer
miR-302c-3p	TAAGTGCTTCCATGTTTCAGTGG
miR-378a-3p	ACTGGACTTGGAGTCAGAAGGC
miR-103a-3p	AGCAGCATTGTACAGGGCTATGA
miR-20a-5p	TAAAGTGCTTATAGTGCAGGTAG
miR-20b-5p	CAAAGTGCTCATAGTGCAGGTAG
miR-106a-5p	AAAAGTGCTTACAGTGCAGGTAG
miR-25-3p	CATTGCACTTGTCTCGGTCTGA
miR-148a-3p	TCAGTGCACTACAGAACTTTGT
miR-92a-3p	TATTGCACTTGTCCCGG
miR-302d-3p	TAAGTGCTTCCATGTTTGAGT
miR-302b-3p	TAAGTGCTTCCATGTTTTAGTAG
miR-302a-3p	TAAGTGCTTCCATGTTTTGGTGA
miR-302a-5p	TAAACGTGGATGTACTTGCTT
miR-93-5p	CAAAGTGCTGTTCGTGCAGGTAG
miR-17-5p	CAAAGTGCTTACAGTGCAGGTA
miR-221-3p	AGCTACATTGTCTGCTGGGT
miR-21-5p	TAGCTTATCAGACTGATGTTGA
miR-183-5p	TATGGCACTGGTAGAATTCACT
miR-363-3p	AATTGCACGGTATCCATCTGTA
miR-182-3p	TTTGGCAATGGTAGAACTCAC

### Western blotting

Total protein was extracted from cells and tissues using radioimmunoprecipitation assay lysis buffer and the protease inhibitor phenylmethylsulfonyl fluoride. Proteins were separated on 10% sodium dodecyl sulfate–polyacrylamide gel electrophoresis gels and electro-transferred onto polyvinylidene fluoride membranes (Millipore, Billerica, MA, USA). Membranes were blocked with 5% (w/v) non-fat dry milk for 1 h followed by an overnight incubation at 4 °C with primary antibodies: Collagen I (1:1000; Abcam), α-SMA (1:2000; Cat. No. 14395-1-AP, Proteintech), Fibronectin (1:1000; Abcam), Thbs2 (1:1000; Cat. No. ab84469, Abcam), and glyceraldehyde 3-phosphate dehydrogenase (GAPDH) (1:10,000; Cat. No. 10494-1-AP, Proteintech). Subsequently, membranes were incubated for 1 h with HRP-conjugated secondary antibodies at room temperature. Finally, bands were detected using an enhanced chemiluminescence kit (Cat. No. P90120, Millipore).

### RNA sequencing

Total RNA was extracted from the right middle lung lobe (3 mice per group) using TRIzol, and RNA quality was measured with a NanoDrop 2000 (ThermoFisher). The isolated RNA was further purified by DNase I treatment and rRNA removal. Library construction and RNA sequencing were then performed on a Novaseq platform.

### Read mapping and differentially expressed gene analysis

The raw data adapters were removed and were clustered with SOAPnuke and Trimmomatic software using the default parameters [27]. Reads were then mapped to the reference genome (Mus_musculus.GRCm39.dna.toplevel.fa) using HISAT2 software (https://daehwankimlab.github.io/hisat2/) [28]. For downstream analysis, gene expression levels were obtained using HTSeq software [29], and the DEGs between two groups were assessed by DEGseq [30] with the following thresholds: fold change ≥ 2 and adjusted *P* value ≤ 0.001. The identified DEGs for each pair were aligned to terms and pathways in the Gene Ontology database (http://geneontology.org/) and Kyoto Encyclopedia of Genes and Genomes database (https://www.kegg.jp/). Significant enrichment was defined as a *P* value of ≤ 0.05.

### Detection of apoptosis and reactive oxygen species

A YO-PRO-1/PI apoptosis and necrosis detection kit (Cat. No. C1075, Beyotime) was used to detect cell apoptosis according to the manufacturer’s instructions. YP1/PI detection buffer was prepared by adding 1 µL YP1 and 1 µL PI into 1 mL basal DMEM/HD. One day after BLM or hESC-exo treatment, cells were incubated for 20 min with 500 µL detection buffer, in the dark and at 37 °C. Cells were then collected and analyzed by flow cytometry.

2’,7’-Dichlorofluorescin diacetate (DCFH-DA; Cat. No. D6883, Sigma) was used to detect ROS. One day after BLM or hESC-exo treatment, cells were incubated for 40 min with 10 µM DCFH-DA, in the dark and at 37 °C. Cells were then collected and analyzed by flow cytometry.

### Statistics

All values are expressed as the mean ± standard error. Differences in survival rates were analyzed using the Log-rank (Mantel-Cox) test; differences in other data types were analyzed by one-way analysis of variance followed by Dunnett's multiple comparisons test, using GraphPad Prism 8 (GraphPad Software Inc., CA, USA). Levels of statistical significance were defined as follows: *P < 0.05, **P < 0.01, and ***P < 0.001.

## Results

### hESC-exo treatment alleviated BLM-induced lung inflammation and improved lung function

hESC-exo were identified as previously described, and characterized by morphology, particle size, and surface markers. Transmission electron microscopy revealed that the exosomes were typically spindle-shaped (Additional file 1: Fig. S1A), and dynamic light scattering assays revealed a mean diameter of 122.7 nm (Additional file 1: Fig. S1B). Western blot analysis revealed that the purified exosomes carried positive markers, e.g., CD63, tumor susceptibility gene 101 (Tsg101), and CD9 (Additional file 1: Fig. S1C). In vivo tracing of PKH67-labeled hESC-exo indicated that the volume of exosomes residing in the lungs peaked at 24 h, and then gradually decreased; however, some exosomes were still resident in the lungs at 72 h (Additional file 1: Fig. S1D).

**Fig. 1:**
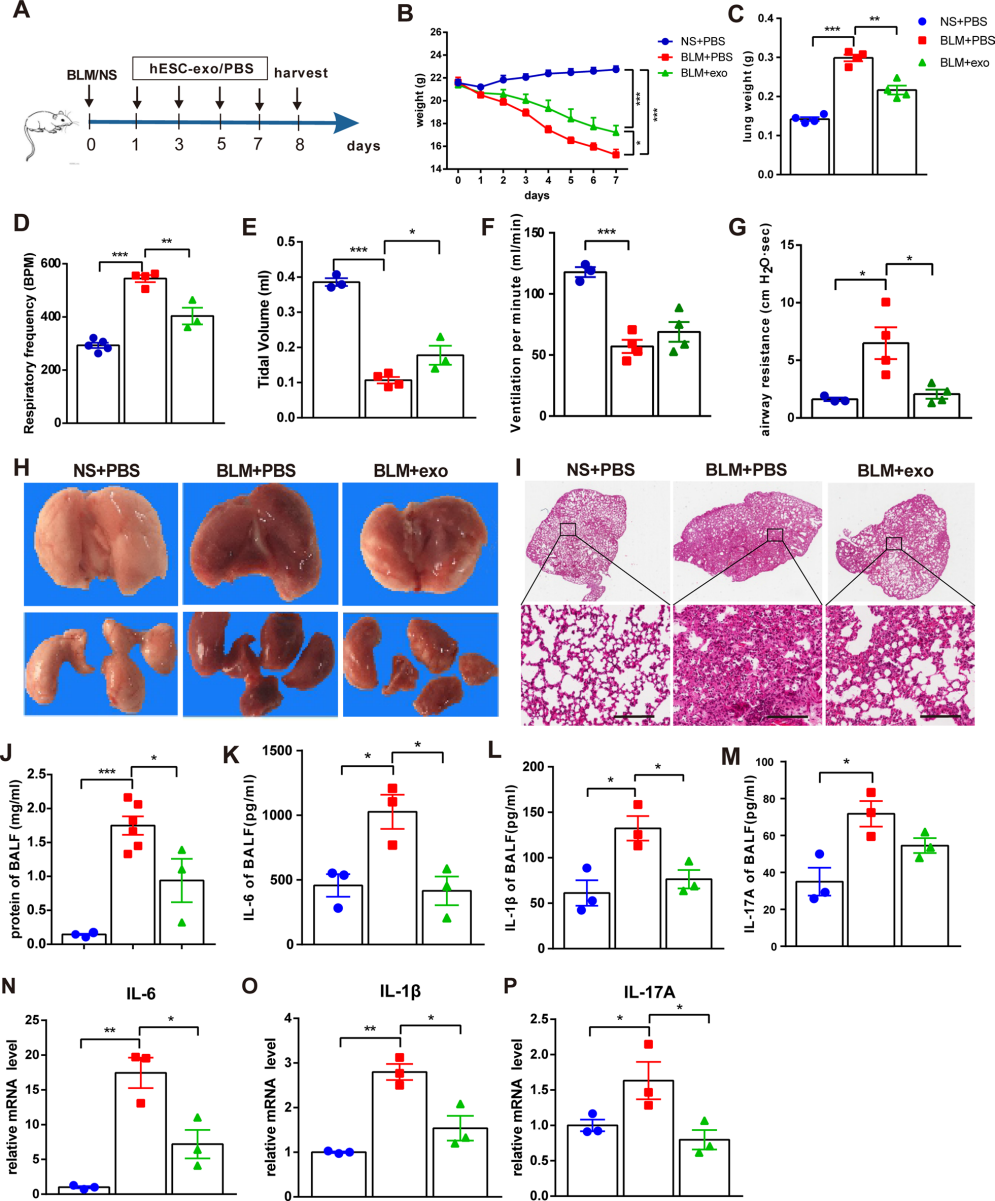
hESC-exo alleviate BLM-induced lung inflammation. **A**. Schema of the experimental procedure. **B**. Mouse body weight changes over the experimental period. **C**. Comparison of lung weights across the three groups of mice. D-G. hESC-exo treatment improved respiratory rate (**D**), tidal volume (**E**), ventilatory volume per minute (**F**), and airway resistance (**G**). H. Lung morphological changes in the three groups. I. Representative lung sections stained with hematoxylin and eosin (**H** and **E**). **J**. Total protein concentration changes in BALF from the three groups of mice. **K**–**M**. The levels of IL-6 (**K**), IL-1β (**L**), and IL-17A (**M**) in BALF from the different groups of mice as determined by ELISA. N-P. qPCR analysis of mRNA levels of the pulmonary inflammatory genes, IL-6 (**N**), IL-1β (**O**), and IL-17A (**P**), in the three groups. Scale bar: 200 μm; *n* = 3–6; mean ± se; *P < 0.05, **P < 0.01, and ***P < 0.001

BLM-induced acute lung injury is mainly characterized by inflammation within one week of the initial insult. To investigate the effect of hESC-exo on BLM-induced inflammation in the lung, BLM-treated mice were administered hESC-exo every two days, with the 8th day after BLM dosing set as the experimental endpoint (Fig. 1A). Weight monitoring identified that hESC-exo treatment significantly alleviated the weight loss caused by BLM toxicity; however, it did not restore the weights of BLM-treated mice to those in the non-BLM-treated control group (Fig. 1B). Moreover, BLM-induced fibrosis notably increased lung weights compared to those in control animals, a condition that also improved after hESC-exo administration (Fig. 1C). In terms of pulmonary function, BLM toxicity remarkably impaired normal breath activities, decreasing respiratory frequency (breaths per minute; BPM), tidal volume (TV), lung minute volume (LMV), and airway resistance (AR). Of these, hESC-exo intervention effectively improved the BPM, TV, and AR indices (Fig. 1D–G). Histological analysis revealed that BLM toxicity directly resulted in gross hemorrhagic necrosis (Fig. 1H), destruction of alveoli structure, and inflammatory infiltration (Fig. 1I). The bronchoalveolar lavage fluid (BALF) of BLM-treated mice was also characterized by elevations of both total protein concentrations and inflammatory cytokines, including interleukin (IL)-6, IL-1β, and IL-17A, compared to that from control mice (Fig. 1J–M). A similar increase in inflammatory cytokines was observed in fibrotic lung tissue at the mRNA level (Fig. 1N–P). All the above pathologies were corrected by hESC-exo administration, with remodeling of the collapsed alveolar architecture, and attenuation of the increased levels of inflammatory cytokines (Fig. 1I–P).

### hESC-exo administration ameliorated BLM-induced pulmonary dysfunction

**Fig. 2:**
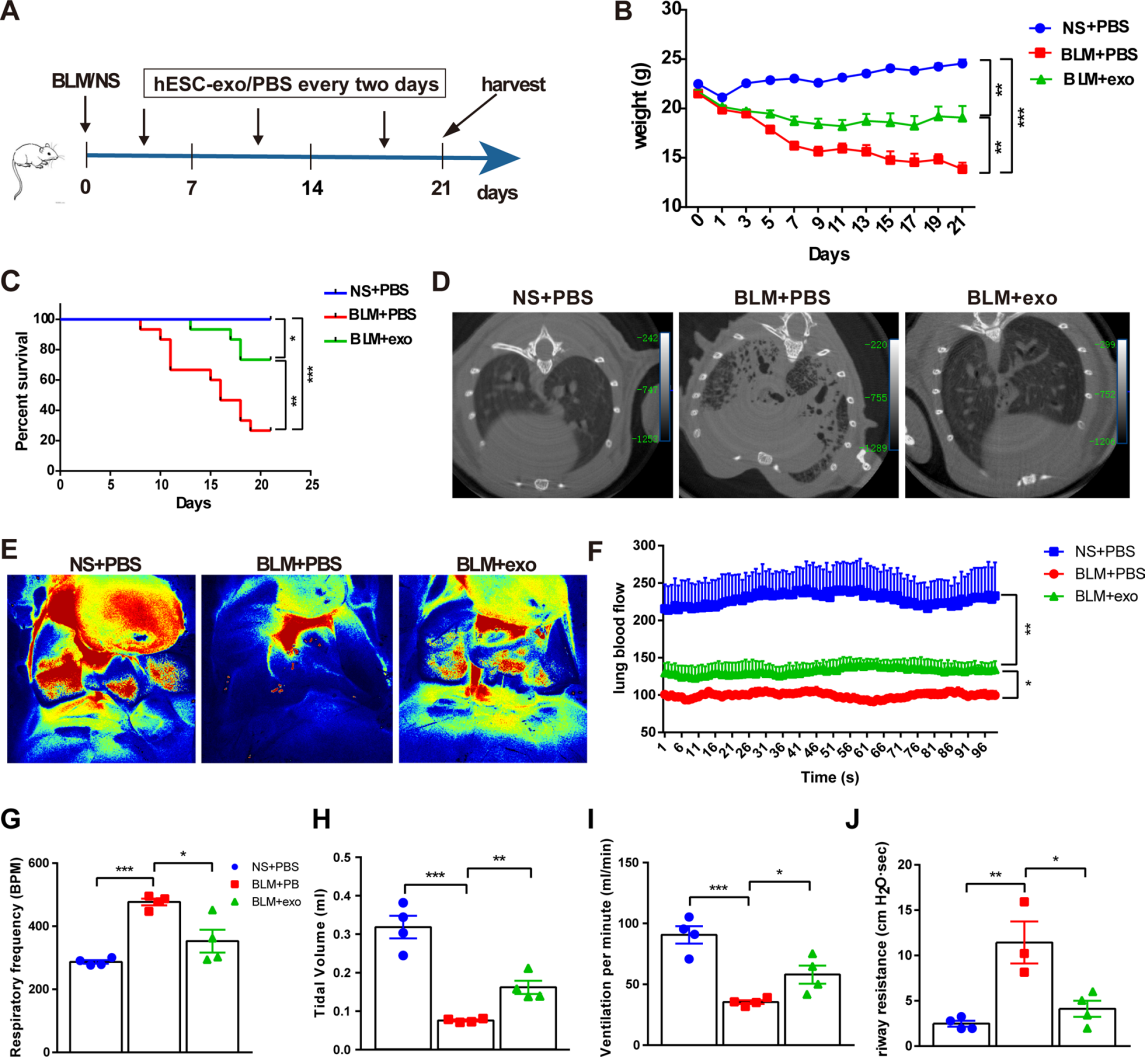
hESC-exo alleviate BLM-induced lung fibrosis in mice. **A**. Schematic of the BLM mouse model preparation and hESC-exo administration procedures. **B**. Mouse body weight changes over the experimental period. **C**. Survival of mice treated with or without hESC-exo throughout the experiment. **D**. Representative CT scan images. The images clearly show that hESC-exo treatment significantly improved BLM-induced lung fibrosis. **E**–**F**. Thermal imaging of pulmonary arterial flow changes. The results showed that BLM treatment resulted in a significant reduction in pulmonary blood flow, which partially recovered after hESC-exo treatment. G-J. Detection of lung function changes: respiratory frequency (**G**), tidal volume (**H**), ventilation per minute (**I**), and airway resistance (**J**). Scale bar: 200 μm; *n* = 3–15; mean ± se; *P < 0.05, **P < 0.01, and ***P < 0.001

Approximately three weeks after BLM challenge, surviving mice develop typical pulmonary fibrosis. Therefore, we used the 21st day after BLM administration as the experimental endpoint to investigate the effect of hESC-exo on pulmonary fibrosis. Over this period, BLM-treated mice were injected with hESC-exo or phosphate-buffered saline (PBS) every two days (Fig. 2A). Analysis of weight changes over this three-week period identified that the hESC-exo treatment successfully reduced the effects of BLM toxicity in the context of the ongoing weight loss in BLM-treated mice, although weights still did not return to the normal level (Fig. 2B). In addition, hESC-exo notably increased the survival rate of BLM-treated mice from 26.67 to 73.34%, though this was still lower than survival rates in the non-BLM-treated group (Fig. 2C). Computed tomography (CT) and laser speckle imaging clearly depicted the sagittal profile of the lung and pulmonary dynamic blood flow, respectively. These imaging data demonstrated that fibrotic lungs in BLM-treated mice were characterized by thickened bronchial walls, dilated lumen, and honeycomb shading. Sustained exosome dosing protected lungs from BLM toxicity and present basically normal lung imaging morphology (Fig. 2D). Pulmonary fibrosis inevitably affects lung blood flow due to the remodeled vascular network. Laser speckle imaging recorded the hemodynamics during a cardiac cycle, and the results showed that the blood flow was repressed in fibrotic lungs compared to that in healthy lungs; again, hESC-exo treatment was able to rescue the decrease in pulmonary blood flow velocity to a certain extent (Fig. 2E, F). Furthermore, pulmonary function monitoring tests determined that hESC-exo blocked the BLM-induced impairment of lung function over this three-week period, decreasing BPM and AR, and increasing TV and LMV (Fig. 2G-J).

### hESC-exo administration attenuated BLM-induced fibrosis in vivo

**Fig. 3:**
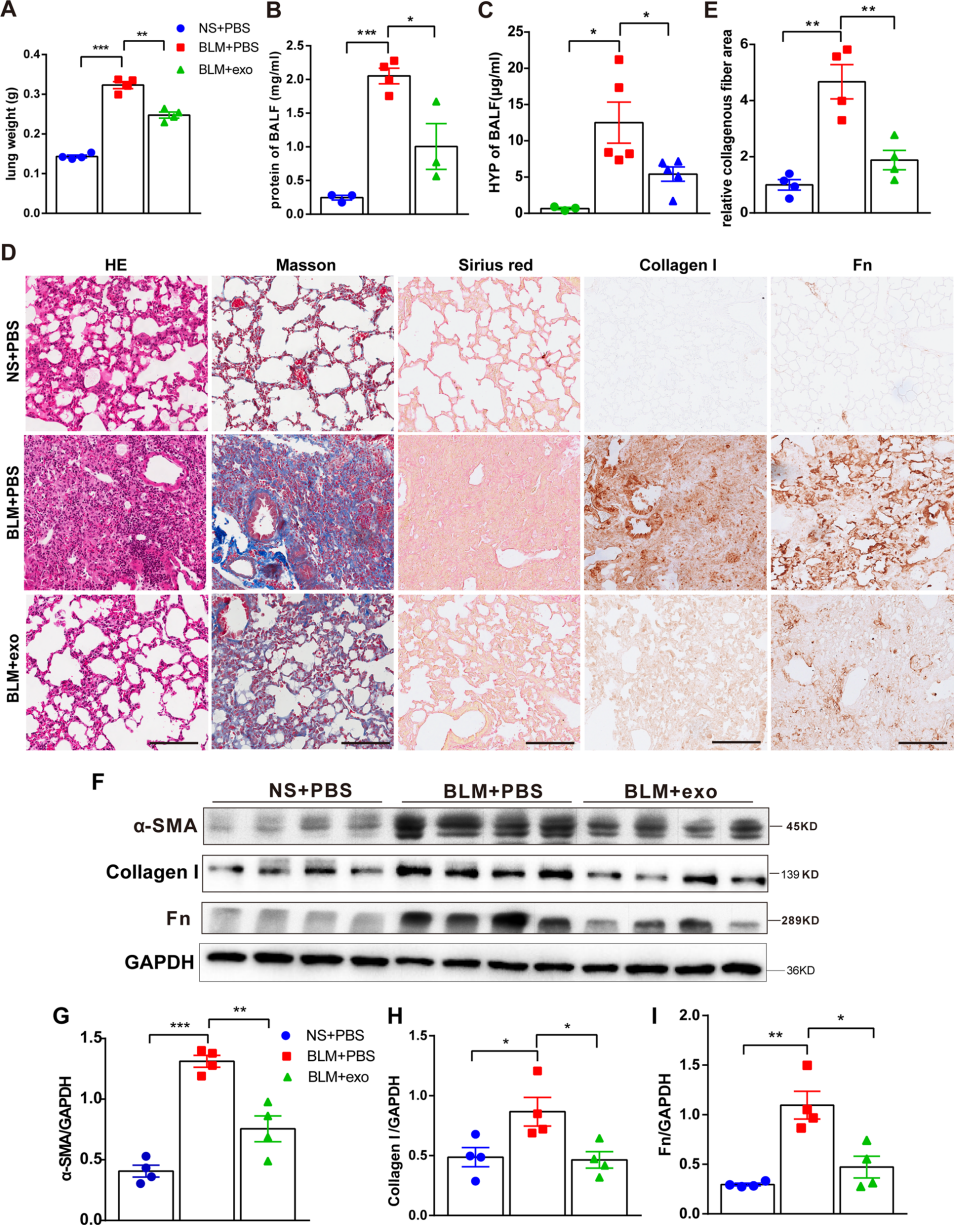
hESC-exo alleviate fibrosis-related destruction of lung structure and function. A-B. Comparison of lung weights (**A**) and BALF total protein concentrations (**B**) in the three groups of mice. **C**. Determination of HYP content in BALF from the three groups of mice. D. Representative images of lung sections stained with hematoxylin–eosin (H&E), Masson’s trichrome and Sirius red, and type I collagen- and fibronectin (Fn)-specific antibodies. E. Relative quantification of the collagenous fiber area indicated by Masson’s trichrome staining in lungs. **F**–**I**. Western blot analysis of α-SMA (**G**), collagen I (**H**), and Fn (**I**) expression in lung tissue lysates. GAPDH was used as a loading control. The blots of α-SMA, Collagen I, Fn and GAPDH were all cropped (**F**) and full-length blots were presented in Additional file 8: Fig. S8. *n* = 3–4; mean ± se; *P < 0.05, **P < 0.01, and ***P < 0.001

BLM-induced pulmonary fibrosis is characterized by the collapse of alveolar structure and increased tissue density, which directly results in increased lung weight. As shown in Fig. 3A, weight gain in the fibrotic lungs was notably higher than that in healthy, non-BLM-treated, lungs. hESC-exo intervention rescued these abnormal lung weights. At the microlevel, TEM revealed that the alveolar epithelial cells of the fibrotic lung showed an abnormal morphology and disorganized layout in BLM-treated mice; alveolar epithelial cells from hESC-exo-treated mice maintained a normal morphology, but failed to recover an organized layout (Additional file 2: Fig. S2). BALF was also collected to analyze fibrosis-associated factors—the total protein and hydroxyproline (HYP) levels. The hESC-exo dosing procedure in our study effectively attenuated the increase in total protein and HYP levels in BLM-treated mice (Fig. 3B, C). At the pathological level, hematoxylin and eosin (H and E) staining depicted the remodeling of lung microstructure, that is, the collapse of alveoli and thickening of airway epithelium in BLM-treated mice. hESC-exo treatment enabled the maintenance of normal lung morphology in the face of BLM toxicity. Masson and Sirius Red staining and the highlighted collagen deposits in the fibrotic lung; hESC-exo administration hindered this accumulation of collagen. Immunohistochemical analysis of collagen I and Fn directly supported the above findings (Fig. 3D, E). The occurrence and development of fibrosis are accompanied by the high expression of specific proteins, such as alpha-smooth muscle actin (α-SMA), collagen I, and fibronectin (Fn). Western blotting and quantitative real-time polymerase chain reaction (qPCR) analysis also highlighted the antifibrotic effect of hESC-exo treatment on α-SMA, collagen I, and Fn (Fig. 3F-I, Additional file 3: Fig. S3 B). Other fibrotic and inflammatory factors have also been assessed in the process of fibrosis such as TGFβ1, IL-6 and IL-1β, but none of them present significant difference (Additional file 3: Fig. S3 A).

### hESC-exo treatment profoundly influenced the gene expression profile in BLM-treated lungs

**Fig. 4:**
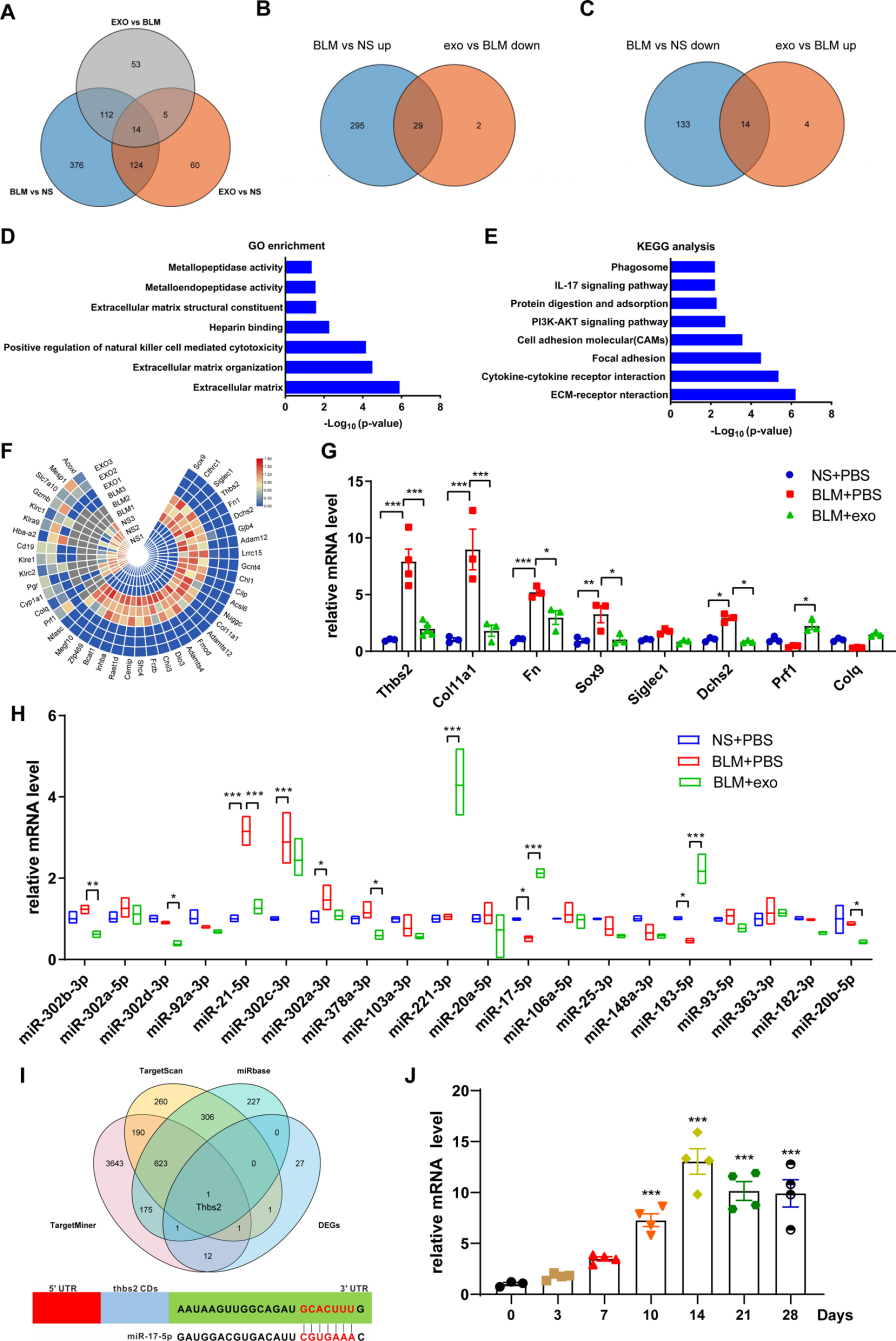
hESC-exo affect the gene expression profile in BLM-induced lungs. **A**. Venn diagram of DEGs identified from comparison of the non-hESC-exo-treated BLM (BLM + PBS) group versus the control group, the hESC-exo-treated BLM (BLM + exo) group vs. the BLM + PBS group, and the BLM + exo group vs. the control group. **B**. Venn diagram of DEGs identified as upregulated in the BLM + PBS group compared to those in the control group, and downregulated in the BLM + exo group in comparison to those in the BLM + PBS group. **C**. Venn diagram of DEGs identified as downregulated in the BLM + PBS group compared to those in the control group, and upregulated in the BLM + exo group in comparison to those in the BLM + PBS group. **D**, **E** GO enrichment and KEGG analysis of the 29 DEGs overlapped of upregulated genes in the BLM + PBS group versus NS + PBS group and downregulated genes in the BLM + exo group versus BLM + PBS group and 14 DEGs overlapped of downregulated genes in the BLM + PBS group vs NS + PBS group and upregulated genes in the BLM + exo group vs BLM + PBS group, totally 43 genes. F. Heat map showing the expression profiles of above 43 DEGs. **G**. To verify the RNA sequencing results, mRNA levels were measured by qPCR. **H**. qPCR analysis to verify the expression profiles of the top 20 microRNAs expressed in hESC-exo in BLM-induced mice. **I**. Venn diagram of target genes of miR-17-5p screened by TargetScan, miRbase, and TargetMiner overlapped with the 43 DEGs. And only the target genes of miR-17-5p had overlap with DEGs that was Thbs2. **J**. Atlas of interaction nodes between miR-17-5p and *Thbs2*. *n* = 3–5; mean ± se; *P < 0.05, **P < 0.01, and ***P < 0.001

RNA-seq facilitated exploration of the mechanism by which hESC-exo combated BLM toxicity in the lung. To screen the key genes for alleviating pulmonary fibrosis of hESC-exo, we focused on investigating differences in the mRNA expression profiles between BLM-treated lungs with or without hESC-exo treatment. Pairwise Venn analysis extracted 29 overlapping upregulated, and 14 overlapping downregulated, genes between the BLM + PBS group and the normal saline (NS) + PBS and BLM + exo groups (Fig. 4A–C). The overlapping differentially expressed genes (DEGs) (29 plus 14), as the key candidate genes for hESC-exo to alleviate pulmonary fibrosis, were subjected to bioinformatics analysis, and the results indicated that they were mainly involved in the ECM, ECM organization, positive regulation of natural killer cell-mediated cytotoxicity, etc., processes that are involved in the following pathways: ECM-receptor interaction, cytokine-cytokine receptor interaction, focal adhesion, cell adhesion molecules, etc. (Fig. 4D, E). A heatmap depicting the mRNA expression of DEGs (Fig. 4F), and qPCR was further performed to confirm the hub genes in the DEGs cluster. From these, *Thbs2*, *Col11a1*, *Fn*, *Sox9*, *Dchs2*, and *Prf1,* emerged as significantly different (Fig. 4G).

miRNAs in exosomes are crucial cargos for regulating crosstalk between cells. In 2004, Mi-Ra et al. first revealed the unique miRNA clusters of hESCs. We assumed that these miRNAs could be packaged into exosomes for intercellular communication. We described the expression patterns of miRNAs in hESC-exo and displayed the partial results in a heatmap (Additional file 4: Fig. S4 A). And qPCR was performed to verify the expression of several high-expressed miRNAs (Additional file 4: Fig. S4 B). We then investigated the expression of top 20 hESC-exo miRNAs in the treated lungs. The results demonstrated that miR-221-3p, miR-17-5p, and miR-183-5p levels were remarkably increased compared to those in the lungs without hESC-exo treatment (Fig. 4H). The target genes of these miRNAs were predicted separately using three open-access miRNA databases, TargetScan, miRbase, and TargetMiner; these genes were then examined to identify if they overlapped with any of the 43 DEGs previously identified, to clarify the specific miRNA and target gene. From the three selected miRNAs, only a target gene of miR-17-5p overlapped with a previously identified DEG, namely Thbs2 (Fig. 4I). The interaction mode of miR-17-5p with Thbs2 is also shown in Fig. 4I. And there are no DEGs overlapped with the target genes of miR-221-3p and miR-183-5p (Additional file 5: Fig. S5A, B). Based on this, we verified the expression level of Thbs2 in vivo at different time points during the progression of BLM-induced IPF. We found that Thbs2 started to show a trend of elevated expression on day 3 after BLM induction, and the level of Thbs2 was significantly higher than that of the control group on day 10, peaked at day 14, which was more than tenfold higher than that of the control group, and continued until day 28 (Fig. 4J).

### hESC-exo treatment alleviated BLM toxicity via the miR-17-5p/Thbs2 axis

Thbs2 is a profibrotic, antiangiogenic matricellular protein that is an attractive target for therapeutic knockdown to combat fibrosis. Based on our findings, we hypothesized that hESC-exo-derived miR-17-5p could directly bind to *Thbs2* to regulate the fibrotic process. In order to verify this hypothesis at the cellular level, we first conducted experiments to test the BLM toxicity on Beas-2b cells. Consistent with the results obtained from the experiments conducted in mouse models, hESC-exo treatment alleviated BLM-induced increased mRNA level of inflammatory factors TGFβ1, IL6, IL-1β, and IL-17A (Additional file 6: Fig. S6A-D), attenuated cellular fibrosis via reducing the increased content of HYP and upregulated mRNA level of collagen I, Fn and Thbs2 induced by BLM (Additional file 6: Fig. S6E-H) in Beas-2b cells. Western blot experiment demonstrated that proteins and factors, including α-SMA, collagen I, Fn and Thbs2, related to fibrosis formation were increased by BLM, and the increases in the proteins and factors were alleviated by hESC-exo treatment (Additional file 6: Fig. S6I-M).

**Fig. 5:**
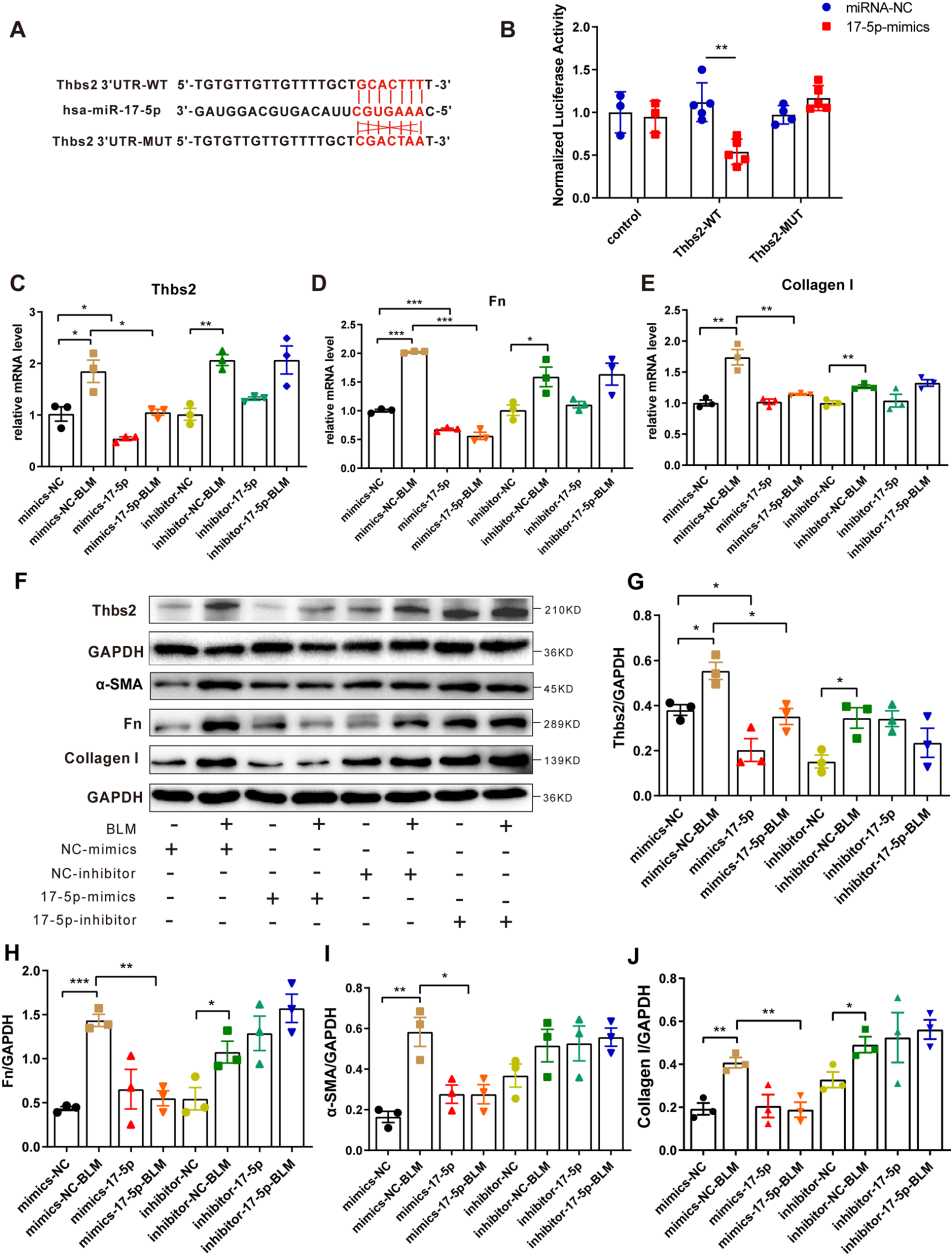
The miR-17-5p/Thbs2 axis downregulates mRNA and protein levels of fibrosis-related genes. **A**. Diagram of the complementary pairing of miR-17-5p with the 3'UTR of the wild-type *Thbs2*, and its inability to interact with the *Thbs2* mutant. **B**. The interaction between miR-17-5p and *Thbs2* was verified by a double luciferase reporter gene assay. C-E. qPCR analysis of the fibrosis-related genes, *Thbs2* (**C**), Fn (**D**), and Col1a1 (**E**), in Beas-2b cells supplemented with NC/miR-17-5p-mimics or NC/miR-17-5p-inhibitor, with or without BLM. **F**. Western blot analysis of the protein levels of fibrosis-related factors, including Thbs2, Fn, α-SMA, and collagen I, in Beas-2b cells supplemented with NC/miR-17-5p mimics or NC/miR-17-5p-inhibitor, with or without BLM. G-J. Statistical analysis of the expression levels of Thbs2 (**G**), Fn (**H**), α-SMA (**I**), and collagen I (**J**). The blots of Thbs2, GAPDH, α-SMA, Fn and Collagen I were all cropped (**F**), and full-length blots and the original blots generated alternative repeats were presented in Fig. S8. The cropped position of the gels is shown in the blank box. *n* = 3–5; mean ± se; *P < 0.05, **P < 0.01, and ***P < 0.001

**Fig. 6:**
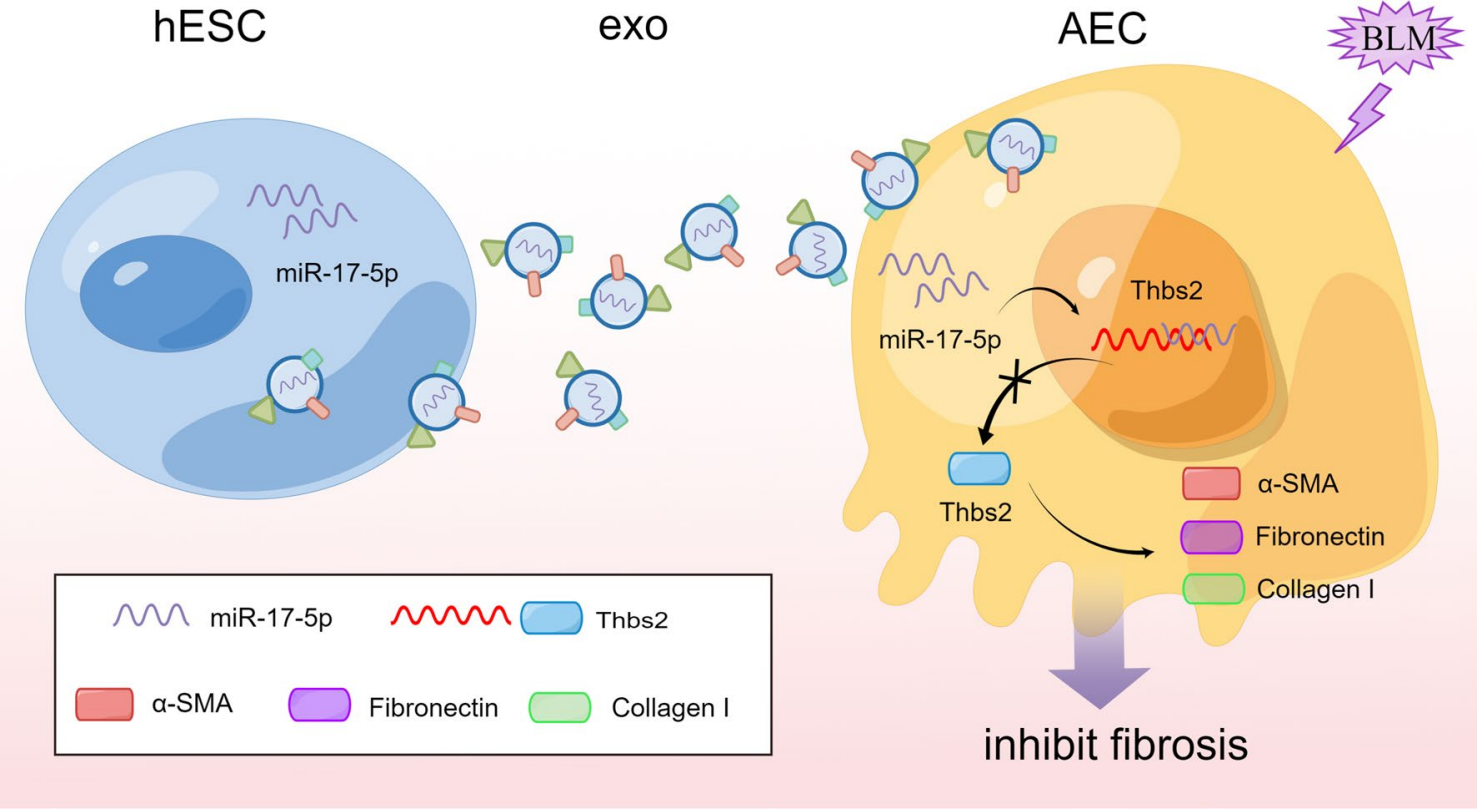
The mechanism of hESC-exo protects against BLM-induced pulmonary fibrosis. miR-17-5p was delivered by hESC-exo into recipient cells like AECs to regulate the mRNA transcription of Thbs2 in nucleus, resulting in the decreased translation of Thbs2 in the cytoplasm. Downstream fibrosis-related proteins like α-SMA, Fn, and Collagen I were downregulated due to the inhibited Thbs2, subsequently

Next, to investigate whether miR-17-5p suppresses *Thbs2* directly through its putative binding site within the 3′ untranslated region (UTR) of *Thbs2*. We constructed a plasmid in which the 3’ UTR, with either a wild-type or mutated miR-17-5p binding site, was inserted downstream of a luciferase reporter (Fig. 5A). Co-transfection of the luciferase reporter and miRNA-17-5p mimics into Beas-2b cells led to the downregulation of luciferase expression (Fig. 5B). qPCR analysis revealed that the BLM-induced increase in *Thbs2* expression could be repressed by miR-17-5p overexpression in Beas-2b cells (Fig. 5C). Correspondingly, the proteins downstream, and regulated by, Thbs2—Fn and collagen I—also changed significantly at the mRNA level, downregulated in Beas-2b cells overexpressing miR-17-5p, whether treated with BLM or not. Overexpression of an inhibitor of miR-17-5p completely blocked these changes (Fig. 5D, E). Western blot analysis further clarified that the BLM-induced increase in Thbs2 protein expression could be antagonized by miR-17-5p overexpression in Beas-2b cells. Other fibrosis-associated proteins, such as α-SMA, Fn, and collagen I, also underwent similar changes (Fig. 5F–J). These results suggest that hESC-exo could deliver miR-17-5p into recipient cells, such as alveolar epithelial cells, to regulate mRNA transcription of *Thbs2* in the nucleus, resulting in decreased translation of Thbs2 in the cytoplasm. Downstream proteins related to fibrosis—α-SMA, Fn, and collagen I—would then be downregulated due to reduced expression of Thbs2 (Fig. 6).

## Discussion

IPF is a chronic, progressive lung disease characterized by progressive lung scarring and a histological picture of usual interstitial pneumonia. Inflammation and fibrosis are the two major driving factors of IPF [2, 31, 32]. In this study, we demonstrated that hESC-exo administration significantly reduced inflammation and fibrosis in BLM-treated animals. The pulmonary response to BLM toxicity mainly consisted of acute inflammation approximately one week after challenge, and progressive fibrosis approximately three weeks after challenge. hESC-exo administration effectively blocked the BLM-induced inflammation trigger and inhibited progression of fibrosis. RNA-seq data indicated that hESC-exo were mainly implicated in the regulation of ECM activities and inflammatory responses in fibrotic lungs. In the case of internal drivers, hESC-exo-derived miR-17-5p repressed the occurrence and development of fibrosis by targeting *Thbs2*, further inhibiting the expression of downstream fibrosis-related genes.

Recently, stem cell-derived exosomes, particularly MSC-exo, have been reported to be therapeutically efficacious in various preclinical studies associated with antifibrosis, and they are now being investigated for clinical translation [33, 34]. The positive effects of MSC-exo in preventing and treating pulmonary fibrosis have been confirmed [35–37]; indeed, they have been highlighted as a potential therapy in SARS-Cov-2-induced pneumonia and pulmonary fibrosis (Clinical Trials.gov identifiers: NCT04276987, NCT04491240, and NCT04493242). However, the role of ESC-exo in lung impairment has not yet been clarified. In this study, we report for the first time the preventive effects of hESC-exo in BLM-induced pulmonary fibrosis. All investigations indicated that hESC-exo notably defeated the BLM-induced inflammatory response and fibrogenesis by repressing cytokine generation and collagen deposition, respectively. hESC-exo administration also improved the survival rate of BLM-treated mice from 26.67 to 73.34%. CT and laser speckle imaging were used to illustrate the sagittal structure and monitor blood flow of lungs. These analyses identified that hESC-exo administration promoted remodeling of the pulmonary vascular architecture and restoration of blood flow. In summary, we comprehensively assessed the preventive effects of hESC-exo on BLM-induced pulmonary fibrosis.

Exosome-carried miRNAs are core regulatory elements in intercellular communication. Houbaviy et al.[38] and Suh et al. [39] pioneered the determination of the miRNA profiles of undifferentiated and differentiated mouse embryonic stem cells. A unique set of miRNAs from hESCs was then identified by cDNA cloning and sequencing. These ESC-specific and highly expressed miRNAs form a complicated network regulating proliferation, differentiation, and metabolism. We hypothesized that some of these ESC-specific miRNAs could be implicated in exosomal intercellular cross-talk. Based on this, a miRNA cluster in hESC-exo was identified, of which the top enriched miRNAs were miR-92a-3p, miR-302, miR-222-3p, miR-17-5p, miR-221-3p, and miR-21-5p. In particular, hESC-exo-derived miR-17-5p played a crucial role in ameliorating the effects of BLM toxicity. Our findings therefore demonstrated that hESC-exo exerted a protective effect against BLM toxicity through the delivery of miR-17-5p, resulting in the suppression of inflammation and fibrosis. These data indicate that miR-17-5p may act as an important antifibrotic, noncoding-RNA, modulator of IPF and other fibrotic diseases. Nevertheless, other issues should be taken into consideration in future, such as the importance of miR-17-5p in hESC-exo-treated IPF relative to other factors, the preventive effects of miR-17-5p in vivo, and the precise molecular mechanism underlying hESC-exo protection against IPF (Additional file 7).

Thbs2 is a disulfide-linked homotrimeric glycoprotein that mediates cell-to-cell, and cell-to-matrix, interactions. Accordingly, Thbs2 is deeply involved in the proliferation, migration, and invasion of tumor cells [40–42]. Transcriptional regulation of *Thbs2* is also associated with several fibrotic diseases, such as liver and cardiac fibrosis. Repression of *Thbs2* greatly contributes to the reduction of fibrosis in the context of pulmonary artery hypertension-induced cardiac injury, indicating the antifibrotic potential of Thbs2 [43–45]. RNA-seq data from our study highlighted that *Thbs2* was involved in the progression of IPF. Repression of *Thbs2* was accompanied by downregulation of fibrosis-associated proteins, including α-SMA, Fn, and collagen I. Our study is the first to identify that Thbs2 may be a potential target in the treatment of pulmonary fibrosis. More importantly, we identified that hESC-exo-derived miR-17-5p directly targeted *Thbs2* to suppress pulmonary fibrosis by hindering collagen deposition. Next, the therapeutic effect of hESC-exos on pulmonary fibrosis models were also under assessment. Results revealed that hESC-exos partly rescued the weight loss caused by BLM toxicity (Additional file 8: Fig. S8 A). In particular, lung micrographs indicated hESC-exos remodeled the collapsed lung architecture and cleared the deposited collagen (Additional file 8: Fig S8 B and C).

Overall, our findings are the first to demonstrate the potential beneficial effects of hESC-exo administration in the BLM-induced IPF model. Importantly, the present study suggests that miR-17-5p-loaded hESC-exo prevent pulmonary fibrosis by directly binding to *Thbs2*, which may offer a new therapeutic strategy for IPF, a disease currently lacking effective treatment.

## Conclusion

In summary, our study indicates that hESC-exo exhibit anti-inflammatory and anti-fibrotic effects in BLM-exposed lungs and the underlying mechanism is via the miR-17-5p/Thbs2 axis. Our results suggest that hESC-exo-based therapy may be a promising new treatment for fibrosis-associated diseases.

### Supplementary Information

**Additional file 1**.** Figure S1**: Identification of hES-exo and retention in lung. A. The image of morphology of exosomes under electron microscope. B. Diameter of hESC-exo identified by dynamic light scattering. C. The CD63, Tsg101, CD9 and GAPDH expression level of hESC cell lysate, hESC-exo and cell supernatant. D. The retention of hESC-exo stained with PKH67 in lung of 24 h, 48 h and 72 h after exo injection via tail vein compared with control. The blots of CD63, Tsg101, CD9 and GAPDH were all cropped (C) and full-length blots were presented in Fig. S9. Scale bar: 500 nm.

**Additional file 2**. **Figure S2**: Ultrastructure of the lungs in 21-day mice. Electron microscopy images of lungs show the collapse of AECs in BLM-treated mice, and ordered arrangement of AECs in hESC-exo-treated mice.

**Additional file 3**. **Figure S3**: Expression of inflammatory genes and fibrosis-related genes in 21-day mice. A, B. qPCR analysis the mRNA levels of pulmonary inflammatory genes of TGFβ1, IL-6, IL-1β and fibrosis-related genes of α-SMA, Fn and Col1a1 among the three groups. Scale bar: 5 μm, n = 3-4, Mean ± se, *p < 0.05, **p < 0.01 and ***p < 0.001.

**Additional file 4**. **Figure S4**: The expression profiles of microRNAs in hESC-exo. A. The expression profiles of a portion of microRNAs of hESC-exo. B. To verify the sequencing results, microRNA levels were measured by real-time qPCR. n= 3, Mean ± se.

**Additional file 5**. **Figure S5**: Screen of target genes of miR-221-3p and miR-183-5p. A. Venn diagram of target genes of miR-221-3p screened by TargetScan, miRbase, and TargetMiner overlapped with the 43 DEGs. B. Venn diagram of target genes of miR-183-5p screened by TargetScan, miRbase, and TargetMiner overlapped with the 43 DEGs.

**Additional file 6**. **Figure S6**: hESC-exo treatment attenuates BLM-induced inflammation and fibrosis of Beas-2b cells. A-D, qPCR analysis the mRNA levels of inflammation-related genes including TGFβ1 (A), IL-6 (B), IL-1β (C) and IL-17A (D). E, Assessment of the HYP content using a HYP measure kit. F-H. qPCR analysis the mRNA levels of fibrosis-related genes including Col1a1 (F), Fn (G) and Thbs2 (H). I-M. Lysates of Beas-2b were subjected to western blotting to determine the expression levels of α-SMA (J), Fn (K), Collagen I (L) and Thbs2(M). GAPDH was used as a loading control. The blots of α-SMA, Fn, GAPDH, Collagen I and Thbs2 were all cropped (I) and full-length blots were presented in Fig. S9. n = 3-4, Mean ± se, *p < 0.05, **p < 0.01 and ***p < 0.001.

**Additional file 7**. **Figure S7**: The hESC-exos administration rescued the BLM-induced pulmonary fibrosis. A. The dynamic monitoring of body weight before and after hESC-exos administration. Arrow, the time point of hESC-exos administration. B. Representative micrographs of lung sections stained with hematoxylin-eosin (H&E), Masson’s trichrome and Sirius red. C. Relative quantification of the collagenous fiber area indicated by Masson’s trichrome staining in lungs. Scale bar: 200 μm; n = 3-5; mean ± se; *P < 0.05 and **P < 0.01.

**Additional file 8**. **Figure S8**: Full-length blots and original blots generated alternative repeats of Western blotting analysis of Figure 3F and Figure 5F.

**Additional file 9**. **Figure S9**. Full-length blots of Western blotting analysis of Figure S1C and Figure S6I.

## Data Availability

The RNA sequencing data generated by this study have been deposited in the NCBI Sequence Read Archive (SRA) database (Accession Number: PRJNA984228). All other data generated or analyzed during this study are included in this published article and its supplementary file.

## Abbreviations

IPF: Idiopathic pulmonary fibrosis
BLM: Bleomycin
hESC-exo: Human embryonic stem cell exosomes
qPCR: Quantitative real-time polymerase chain reaction
WB: Western blot
Thbs2: Thrombospondin-2
ECM: Extracellular matrix
TGF-β: Transforming growth factor β
MSCs: Mesenchymal stem cells
MSC-exo: MSC-derived exosomes
TEM: Transmission electron microscopy
Tsg101: Tumor susceptibility gene 101
BPM: Breaths per minute
TV: Tidal volume
LMV: Lung minute volume, AR: airway resistance
BALF: Bronchoalveolar lavage fluid
PBS: Phosphate-buffered saline
CT: Computed tomography
HYP: Hydroxyproline
H&E: Hematoxylin and eosin
α-SMA: Alpha-smooth muscle actin
Fn: Fibronectin
DEGs: Differentially expressed genes
UTR: Untranslated region
DMEM: Dulbecco’s Modified Eagle’s Medium
FBS: Fetal bovine serum
HRP: Horseradish peroxidase
ELISA: Enzyme-linked immunosorbent assay
DCFH-DA: 2′,7′-Dichlorofluorescin diacetate
