# Supplementary figures and images for: Exosomal miR-17-5p from human embryonic stem cells prevents pulmonary fibrosis by targeting thrombospondin-2
Source: Stem Cell Res Ther. 2023 Sep 4;14:234. doi: 10.1186/s13287-023-03449-7 (PMC10478444; doi:10.1186/s13287-023-03449-7)

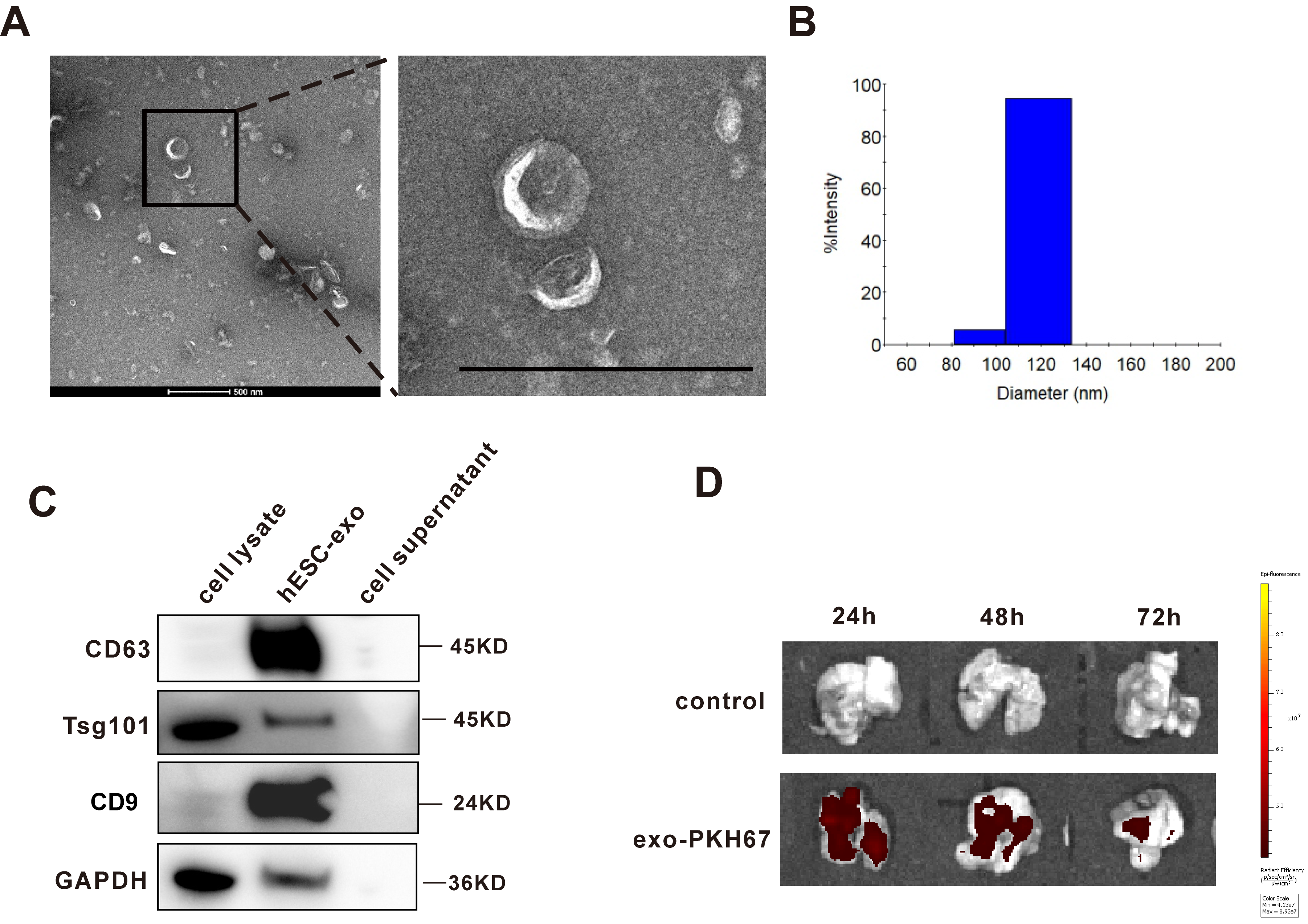

Supplement: Supplementary file 1 — Additional file 1. Figure S1: Identification of hES-exo and retention in lung. A. The image of morphology of exosomes under electron microscope. B. Diameter of hESC-exo identified by dynamic light scattering. C. The CD63, Tsg101, CD9 and GAPDH expression level of hESC cell lysate, hESC-exo and cell supernatant. D. The retention of hESC-exo stained with PKH67 in lung of 24 h, 48 h and 72 h after exo injection via tail vein compared with control. The blots of CD63, Tsg101, CD9 and GAPDH were all cropped (C) and full-length blots were presented in Fig. S9. Scale bar: 500 nm. [file 13287_2023_3449_MOESM1_ESM.tif]

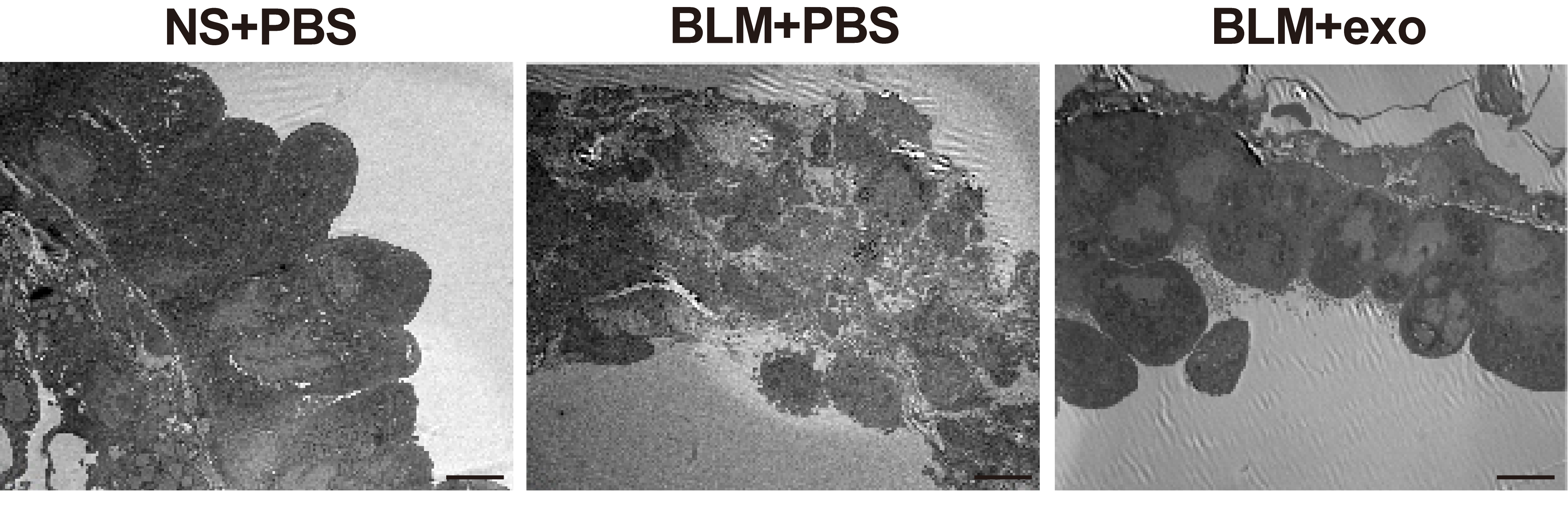

Supplement: Supplementary file 2 — Additional file 2. Figure S2: Ultrastructure of the lungs in 21-day mice. Electron microscopy images of lungs show the collapse of AECs in BLM-treated mice, and ordered arrangement of AECs in hESC-exo-treated mice. [file 13287_2023_3449_MOESM2_ESM.tif]

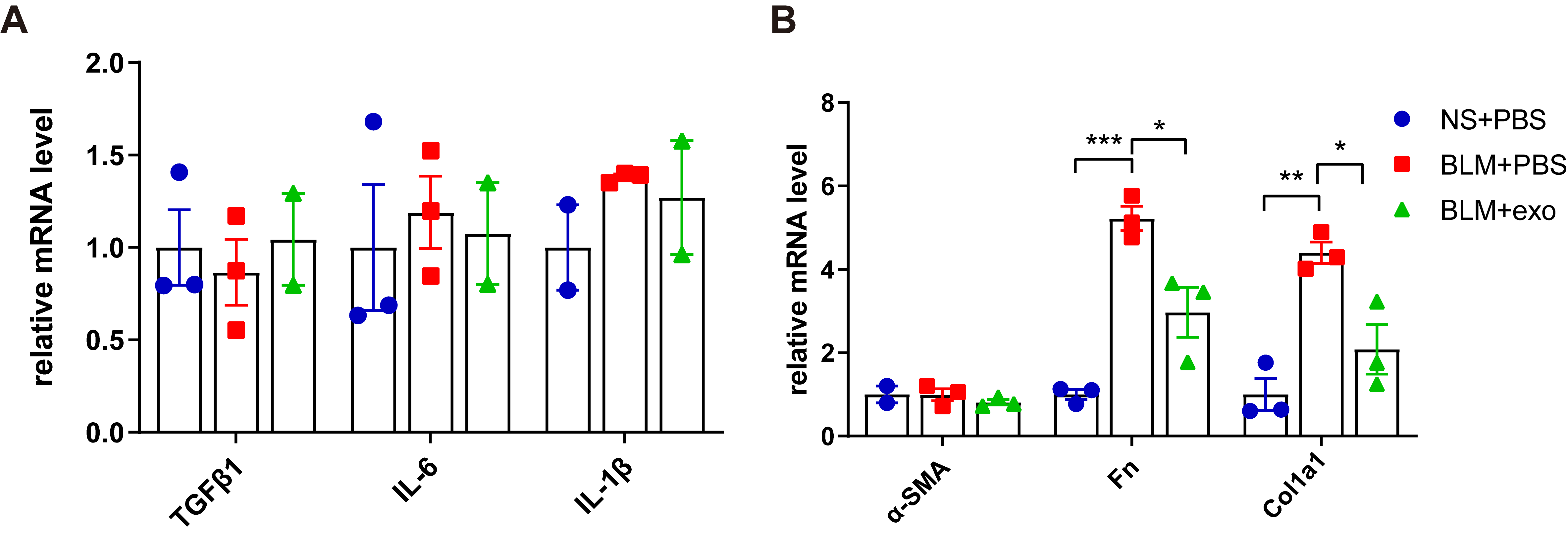

Supplement: Supplementary file 3 — Additional file 3. Figure S3: Expression of inflammatory genes and fibrosis-related genes in 21-day mice. A, B. qPCR analysis the mRNA levels of pulmonary inflammatory genes of TGFβ1, IL-6, IL-1β and fibrosis-related genes of α-SMA, Fn and Col1a1 among the three groups. Scale bar: 5 μm, n = 3-4, Mean ± se, *p < 0.05, **p < 0.01 and ***p < 0.001. [file 13287_2023_3449_MOESM3_ESM.tif]

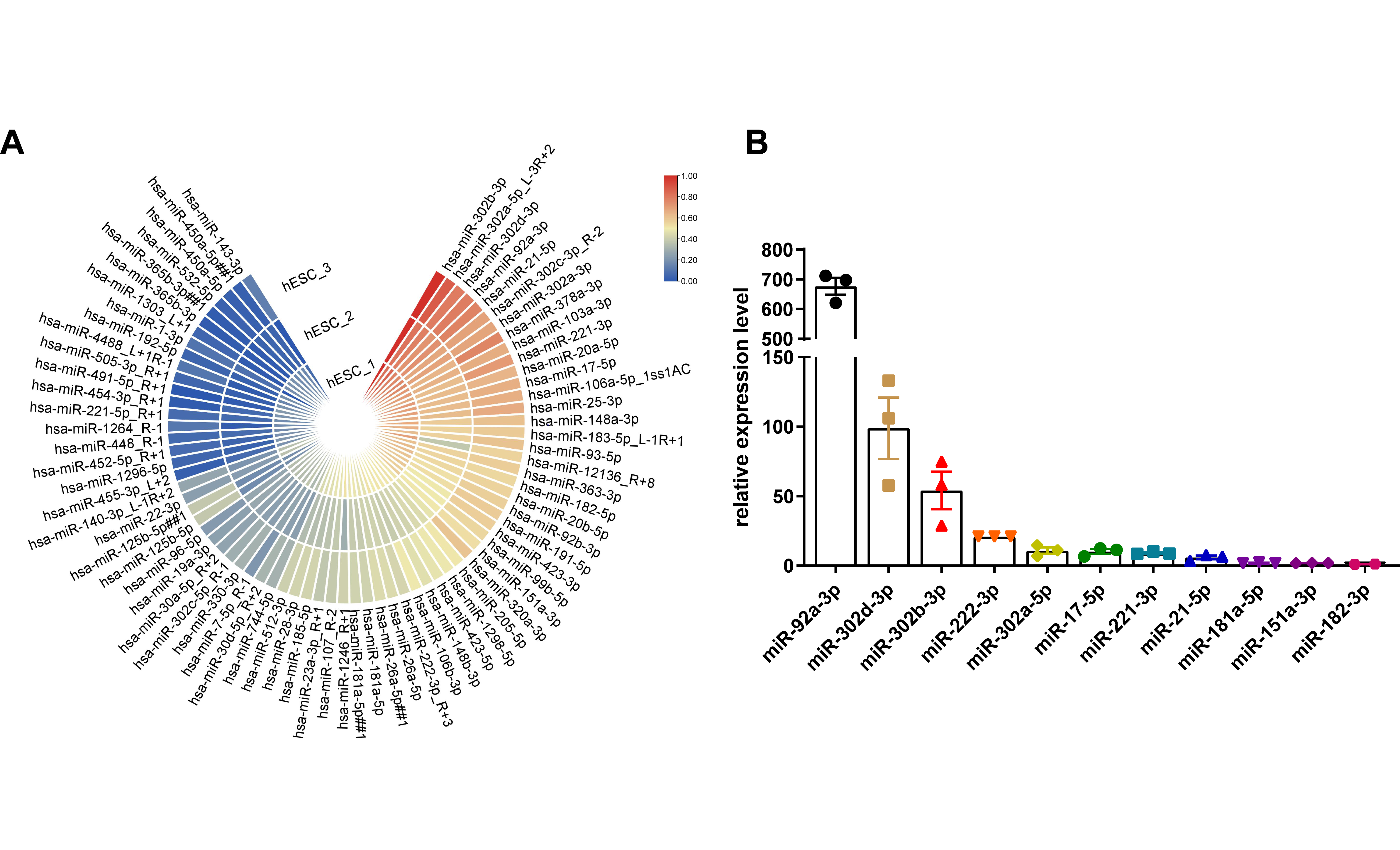

Supplement: Supplementary file 4 — Additional file 4. Figure S4: The expression profiles of microRNAs in hESC-exo. A. The expression profiles of a portion of microRNAs of hESC-exo. B. To verify the sequencing results, microRNA levels were measured by real-time qPCR. n= 3, Mean ± se. [file 13287_2023_3449_MOESM4_ESM.tif]

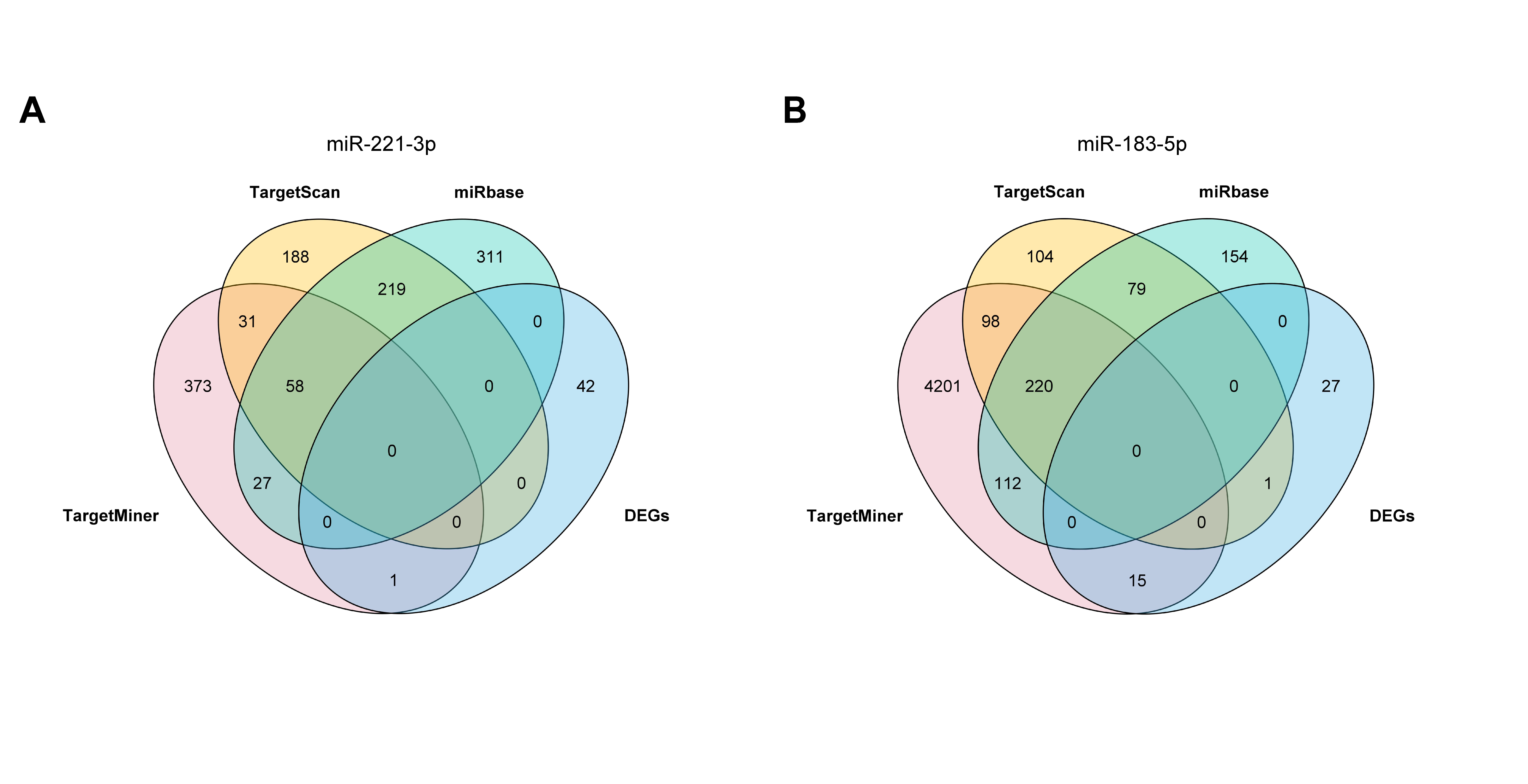

Supplement: Supplementary file 5 — Additional file 5. Figure S5: Screen of target genes of miR-221-3p and miR-183-5p. A. Venn diagram of target genes of miR-221-3p screened by TargetScan, miRbase, and TargetMiner overlapped with the 43 DEGs. B. Venn diagram of target genes of miR-183-5p screened by TargetScan, miRbase, and TargetMiner overlapped with the 43 DEGs. [file 13287_2023_3449_MOESM5_ESM.tif]

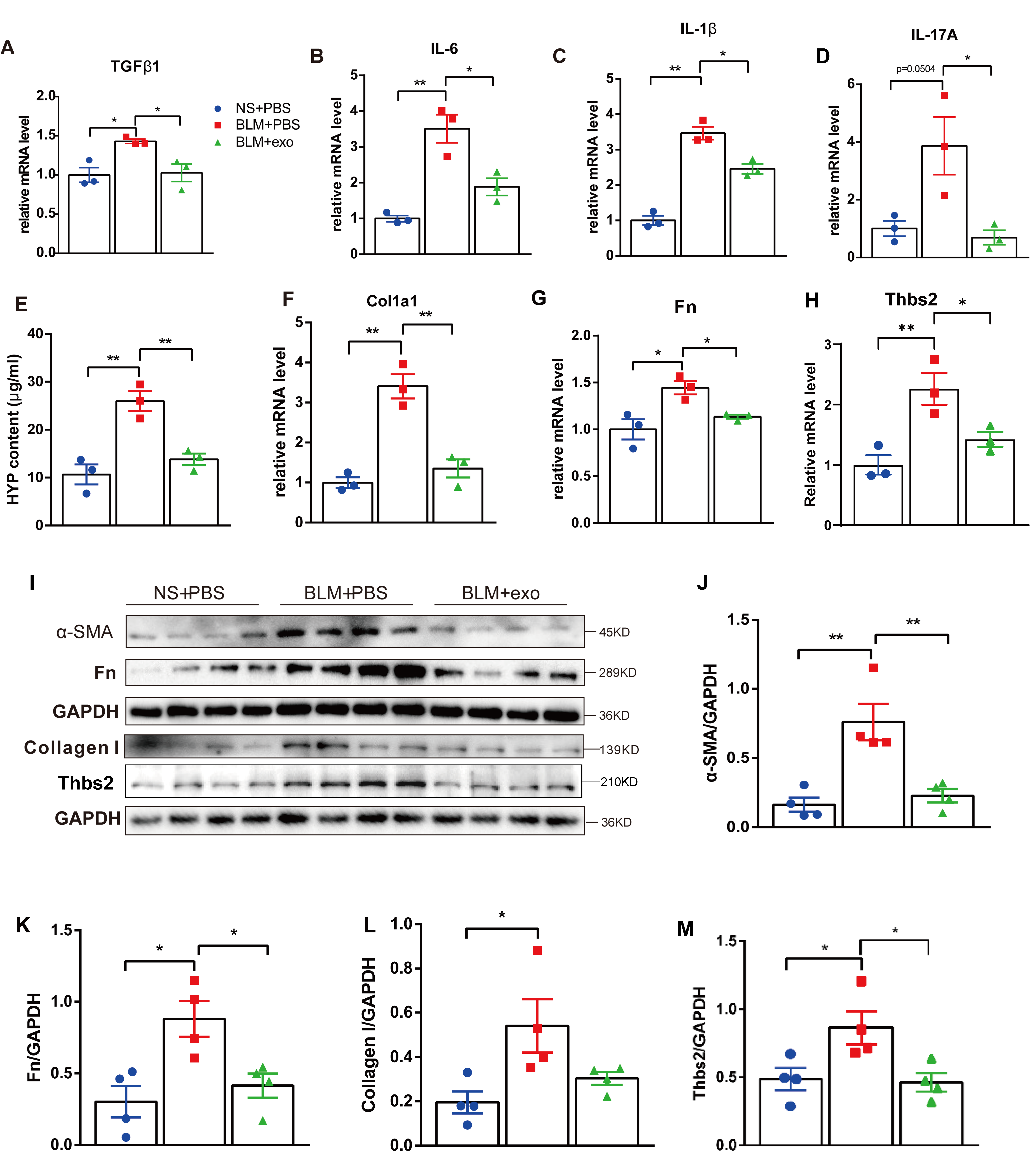

Supplement: Supplementary file 6 — Additional file 6. Figure S6: hESC-exo treatment attenuates BLM-induced inflammation and fibrosis of Beas-2b cells. A-D, qPCR analysis the mRNA levels of inflammation-related genes including TGFβ1 (A), IL-6 (B), IL-1β (C) and IL-17A (D). E, Assessment of the HYP content using a HYP measure kit. F-H. qPCR analysis the mRNA levels of fibrosis-related genes including Col1a1 (F), Fn (G) and Thbs2 (H). I-M. Lysates of Beas-2b were subjected to western blotting to determine the expression levels of α-SMA (J), Fn (K), Collagen I (L) and Thbs2(M). GAPDH was used as a loading control. The blots of α-SMA, Fn, GAPDH, Collagen I and Thbs2 were all cropped (I) and full-length blots were presented in Fig. S9. n = 3-4, Mean ± se, *p < 0.05, **p < 0.01 and ***p < 0.001. [file 13287_2023_3449_MOESM6_ESM.tif]

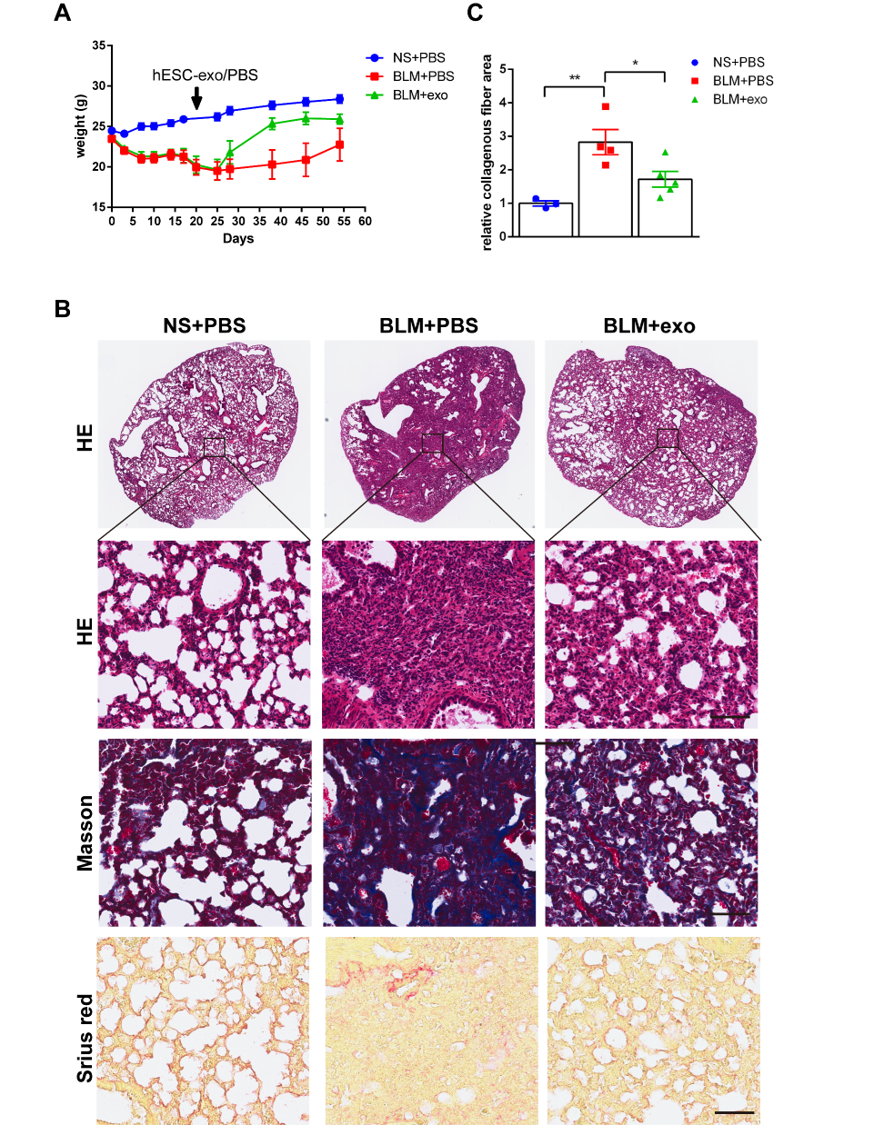

Supplement: Supplementary file 7 — Additional file 7. Figure S7: The hESC-exos administration rescued the BLM-induced pulmonary fibrosis. A. The dynamic monitoring of body weight before and after hESC-exos administration. Arrow, the time point of hESC-exos administration. B. Representative micrographs of lung sections stained with hematoxylin-eosin (H&E), Masson’s trichrome and Sirius red. C. Relative quantification of the collagenous fiber area indicated by Masson’s trichrome staining in lungs. Scale bar: 200 μm; n = 3-5; mean ± se; *P < 0.05 and **P < 0.01. [file 13287_2023_3449_MOESM7_ESM.tif]
